# Melatonin attenuates smoking‐induced hyperglycemia via preserving insulin secretion and hepatic glycogen synthesis in rats

**DOI:** 10.1111/jpi.12475

**Published:** 2018-03-25

**Authors:** Tianjia Li, Leng Ni, Zhewei Zhao, Xinnong Liu, Zhichao Lai, Xiao Di, Zhibo Xie, Xitao Song, Xuebin Wang, Rui Zhang, Changwei Liu

**Affiliations:** ^1^ Department of Vascular Surgery Peking Union Medical College Hospital Chinese Academy of Medical Sciences and Peking Union Medical College Beijing China

**Keywords:** hyperglycemia, insulin, liver glycogen synthase, melatonin, melatonin receptor‐2, smoking

## Abstract

Epidemiology survey indicated that cigarette smoking is a risk factor of diabetes. However, the precise mechanisms remain to be clarified. In this study, we found that smoking caused metabolic malfunctions on pancreas and liver in experimental animal model. These were indicated by hyperglycemia, increased serum hemoglobin A1c level and decreased insulin secretion, inhibition of liver glycogen synthase (LGS), and hepatic glycogen synthesis. Mechanistic studies revealed that all these alterations were caused by the inflammatory reaction and reactive oxygen species (ROS) induced by the smoking. Melatonin treatment significantly preserved the functions of both pancreas and liver by reducing β cell apoptosis, CD68‐cell infiltration, ROS production, and caspase‐3 expression. The siRNA‐knockdown model identified that the protective effects of melatonin were mediated by melatonin receptor‐2 (MT2). This study uncovered potentially underlying mechanisms related to the association between smoking and diabetes. In addition, it is, for first time, to report that melatonin effectively protects against smoking‐induced glucose metabolic alterations and the signal transduction pathway of melatonin is mainly mediated by its MT2 receptor. These observations provide solid evidence for the clinically use of melatonin to reduce smoking‐related diabetes, and the therapeutic regimens are absent currently.

## 1 INTRODUCTION

Cigarette smoking is an unhealthy lifestyle habit. Diabetes is of multifactorial origin and is associated with environmental factors and lifestyle. Notably, diabetes appears to be heavily influenced by smoking,^1^ and clinical epidemiology surveys involving homeostasis model assessment demonstrates that smoking may interfere with blood glucose balance.^2^ However, the mechanisms underlying the effects of smoking on hyperglycemia and β cell function have not yet been investigated. Given the high prevalence of diabetes and its association with smoking, it is of utmost importance to study the intrinsic underlying mechanism and develop therapeutic interventions against this disease.

The pineal hormone melatonin, which is well known for its chronobiotic activity, stimulates coordination of biochemical oscillations by targeting the circadian timing system.^3^, ^4^ Melatonin also has various pleiotropic effects, such as modulation of daily patterns of glucose metabolism and regulation of pancreatic β cell secretion.^5^, ^6^ There is an emerging link between melatonin and glucose metabolism. Reduced melatonin levels are positively associated with increased risk of glycemic disorder,^7^ whereas melatonin administration decreases the risk of glycemic disturbance.^8^ Additionally, melatonin has anti‐inflammatory and antioxidative effects. Our previous study showed that melatonin can attenuate inflammatory and oxidative injury caused by cigarette smoking.^9^, ^10^ Therefore, it is reasonable to hypothesize that melatonin exerts a beneficial influence on glycemic control in smokers. Additionally, melatonin has been shown to relate with glucose transport and gluconeogenesis in liver.^11^

The liver is particularly involved in glucose homeostasis.^12^ High levels of blood glucose are primarily cleared from blood by conversion into glycogen in the liver. Therefore, the restoration of hepatic glycogen ameliorates hyperglycemia. The disturbance of hepatic glycogen accumulation contributes to the development of hyperglycemia.^13^ As a key enzyme in glycogenesis, liver glycogen synthase (LGS), converts glucose into glycogen. The enzyme combines excess glucose into a polymeric chain for storage as glycogen.^14^ Therefore, we want to analyze which effects of hepatic glycogen accumulation and the expression of LGS play in the smoking.

The two functional melatonin receptors, MT1 and MT2, are associated with numerous physiological functions.^15^ Different types of receptors mediate diverse roles. In the vasculature, animal studies suggest that melatonin has dual effects, depending on the specific receptor type activated, with vasoconstriction occurring after MT1‐activation and vasorelaxation after MT2‐activation.^16^ Both receptors, but predominantly the MT2 receptor, are subject to internalization, maintaining homeostasis.^17^ A previous study suggested that the MT2 receptor regulates β cell function; further, deleterious MT2 receptor activity is known to increase diabetes risk.^18^, ^19^ The stimulatory effects of melatonin on insulin secretion rely that the melatonin receptors couple to G proteins mediating a stimulatory effect on insulin secretion.^20^ These results indicate that melatonin may regulate glucose metabolism by binding to receptors.^21^, ^22^, ^23^ We put forward the hypothesis that glucose metabolic effects of melatonin are mediated by receptor binding.

There are few studies of the relationship between smoking and hyperglycemia. Moreover, the effects of melatonin on glucose metabolism have not been thoroughly examined. To resolve these issues, we evaluated pancreatic insulin secretion and hepatic glycogen accumulation in the smoking rat model, and investigated the mechanism underlying the pathogenesis of hyperglycemia. The findings provide novel insight into the potential therapeutic utility of melatonin in glucose regulation.

## 2 METHODS

### 2.1 Animal studies

This study was carried out in accordance with the National Institutes of Health Guide for the Care and Use of Laboratory Animals and the Animal Management Rules of the Chinese Ministry of Health. All experiments were approved by the Animal Care Committee of Peking Union Medical College (No.XHDW‐20150017). Adult male Sprague‐Dawley (SD) rats, with a mean weight of 350 g, were provided and raised by the Laboratory Animal Center of Peking Union Medical College Hospital. Rats were allowed free access to water and standard laboratory chow, and maintained on a 12‐hour light/12‐hour dark cycle in a temperature‐controlled room (25‐28°C).

The rats were randomly divided into four groups (n = 8, per group): (i) normal control group—rats received normal feeding; (ii) melatonin control group—rats received normal feeding and were intraperitoneally injected with melatonin; (iii) smoking group—rats were exposed to cigarette smoke; and (iv) smoking and melatonin group—rats were exposed to cigarette smoke and intraperitoneally injected with melatonin. Rats were exposed to the smoke of 20 cigarettes (11 mg of tar and 0.8 mg of nicotine per cigarette) each day. The period of daily smoke exposure was 120 minutes, between 9:00‐10:00 and 15:00‐16:00, for 3 months. Melatonin (Sigma‐Aldrich Co. LLC, St. Louis, MO, USA) was dissolved in dimethyl sulphoxide and diluted by normal saline, intraperitoneally injected at a dose of 10 mg/kg/day.^10^ After 3 months, the rats were anesthetized by an injection of pentobarbital sodium. The pancreas and liver were isolated and cleaned of surrounding tissue for the following experiments. Blood samples were drawn for further biochemical analyses. Blood glucose was measured by glucometer (Roche, Basel, Switzerland). Blood insulin was measured by enzyme‐linked immunosorbent assay kit (Cusabio, Wuhan, China) according to the manufacturer's instructions.

### 2.2 Cell lines and culture

Rat β cells and hepatocytes (Institute of Basic Medical Sciences, Chinese Academy of Medical Sciences and Peking Union Medical College) were used in this study. β Cells were cultured in RPMI‐1640 basal medium (Hyclone, Logan, UT, USA) with 10% newborn calf serum (Gibco, Carlsbad, CA, USA). Hepatocytes were cultured in MEM‐EBSS basal medium (Hyclone, Logan, UT, USA) with 20% newborn calf serum (Gibco, Carlsbad, CA, USA).

### 2.3 siRNA interference

siRNA was used to knock down the expression of endogenous melatonin receptors. The predesigned siRNA duplexes for MT1, MT2, and the negative control were purchased from GenePharma Co., Ltd. (Shanghai, China). Cells were incubated in 6‐cm dishes until 80% confluent and washed with phosphate‐buffered saline, and the medium was then replaced with 2 mL of fresh basal medium. Transfection was performed with Lipofectamine RNAiMAX Reagent according to the manufacturer's instructions (Invitrogen, Carlsbad, CA, USA), after which the cells were incubated for 24 hours.

### 2.4 Western blot analysis

Proteins extracted from cells or tissues were separated by SDS‐PAGE and then transferred to a polyvinylidene fluoride membrane. Specific proteins were detected using the following antibodies: anti‐β‐actin (1:5000, Sigma‐Aldrich, St Louis, MO, USA), anti‐LGS (1:1000, Santa Cruz Biotechnology, Dallas, TX, USA), cleaved caspase‐3 (1:500, Cell Signaling Technology, Danvers, MA, USA), and antimelatonin receptors (MT1 and MT2) (1:1000; Santa Cruz Biotechnology, Dallas, TX, USA). Bovine serum albumin (5%) in Tris‐buffered saline with Tween 20 was used as the blocking and washing solution. Horseradish peroxidase–conjugated secondary antibodies were incubated with the membrane, and antibody complexes were detected using an electrochemiluminescence kit (Engreen, Beijing, China).

### 2.5 RNA extraction and quantitative real‐time polymerase chain reaction

Total RNA was extracted using TRIzol reagent (Invitrogen, Carlsbad, CA, USA) according to the manufacturer's protocol and quantified using a NanoDrop 2000 spectrophotometer (ThermoFisher Scientific, Bremen, Germany). cDNAs were synthesized from the mRNAs using GenestarScriptRT (Genestar Biotech, Beijing, China). qRT‐PCR was performed in a Bio‐Rad CFX‐96 System (Bio‐Rad, Foster City, CA, USA) using SYBR Premix (Genestar Biotech, Beijing, China). The specific forward (F) and reverse (R) primers used were as follows: MT1, F 5ʹ‐ACCTGCTGGTCATCCTGTCTG‐3ʹ and R 5ʹ‐GCCAAGGGAAATGGGTAAATAG‐3ʹ; MT2, F 5ʹ‐TGAGTGTCATTGGCTCTGTCTTC‐3ʹ and R 5ʹ‐CCAGATGAGGCTGATGTAGAGG‐3ʹ; LGS, F 5ʹ‐AAGAGTTTGTCCGAGGCTGTC‐3ʹ and R 5ʹ‐CCTGCATGAAACACCCGAAAC‐3ʹ; insulin, F 5ʹ‐CCCAAGTCCCGTCGTGAAG ‐3ʹ and R 5ʹ‐CCAGTTGGTAGAGGGAGCAGAT‐3ʹ; and β‐actin, F 5ʹ‐AGCCATGTACGTAGCCATCC‐3ʹ and R 5ʹ‐CTCTCAGCTGTGGTGGTGAA‐3ʹ. Each sample was analyzed in three replicate wells. Relative mRNA expression levels were normalized to the levels of the endogenous reference gene, β‐actin.

### 2.6 Electron microscopy analysis

Electron microscopy is an effective method to study β cell structure and function. During the process of collecting islets for examination by electron microscopy, the pancreas was cut into small pieces (2 mm) and fixed in precooled glutaraldehyde (4°C) for 24 hours. The β cells were studied by transmission electron microscopy to estimate the number of secretory granules.

### 2.7 Histology and pathological staining

The harvested tissues were processed using standard procedures in a series of graded alcohols and xylene and then paraffin‐embedded. Paraffin‐embedded slices were serially sectioned at 4‐mm intervals and mounted on slides. The tissue slides were stained with Periodic acid‐Schiff and alizarin red, to detect hepatic glycogen and vascular calcification, respectively. The staining area was measured using Image‐Pro Plus 6.0 software (Media Cybernetics, Inc., Bethesda, MD, USA). Terminal deoxynucleotidyl transferase dUTP nick end labeling (TUNEL) assay was carried out using the In Situ Cell Death Detection Kit, peroxidase (POD, No. 11772465001; Roche Applied Science, Indianapolis, IN, USA), according to the manufacturer's instructions. The POD is visualized by a precipitating substrate diaminobezidine (DAB).

### 2.8 Immunohistochemistry

Immunohistochemical (IHC) staining was performed on the sectioned tissue to assess the quantity of functional factors. After blocking in phosphate‐buffered saline containing 5% goat serum for 30 minutes at room temperature (20‐25°C), the sections were incubated overnight at 4°C with primary antibodies: insulin, CD68, and caspase‐3 (1:200; Abcam, Cambridge, UK). The following day, signal amplification was performed with horseradish peroxidase–conjugated goat anti‐rabbit IgG secondary antibody (1:500; ZhongshanJinqiao Biotechnology Co., Ltd, Beijing, China). The expression levels of factors were quantified using integrated optical density values generated by Image‐Pro Plus 6.0 software.

### 2.9 Fluorescence staining of reactive oxygen species

Fluorescence staining was performed on the frozen sections of aortas to assess the ROS levels. Islet sections were incubated in phosphate‐buffered saline (PBS) with dihydroethidium (5 μmol/L; Sigma‐Aldrich, St Louis, MO, USA) for 30 minutes at 37°C, and sections were then washed with PBS three times. The nuclei were stained with DAPI (4′,6‐diamidino‐2‐phenylindole). The level of ROS was assessed by fluorescence microscopy. Confocal images of sections were sequentially acquired with Zeiss AIM software on a Zeiss LSM 700 confocal microscope system (Carl Zeiss Jena, Oberkochen, Germany). For quantification, three randomly selected high‐power fields (HPFs) (400× for immunofluorescence studies) were analyzed in each section. The mean red fluorescence intensity per HPF for each rat was then determined.

### 2.10 Statistical analysis

All data are expressed as means ± standard error and were compared by ANOVA and Tukey post hoc test (using SPSS19.0). Statistical significance was defined as *P* < .05.

## 3 RESULTS

### 3.1 Melatonin decreases the smoking‐induced hyperglycemia and preserves insulin secretion

**Figure 1:**
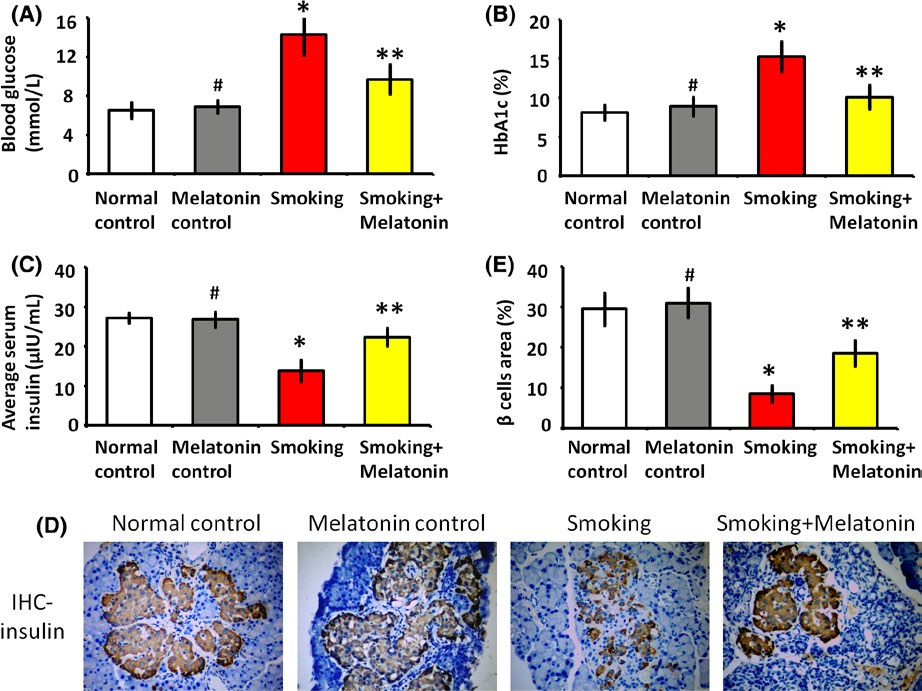
The levels of blood glucose and insulin secretion. (A and B) The level of blood glucose and hemoglobin A1c (HbA1c) in the different groups. (C) Serum insulin levels of the different groups. (D) The brownfield area in the immunohistochemistry (IHC) images shows the area of β cells in the islets. (E) The histogram shows quantification of β cells area in IHC images. #*P* > .05 melatonin control vs normal control, *P < .05 smoking vs normal control, **P < .05 smoking+melatonin vs smoking

After 3 months, the smoking rats had higher blood glucose than the normal control group (Figure 1). Additionally, hemoglobin A1c (HbA1c) was also significantly elevated in serum from smoking rats, reflecting the progression of hyperglycemia. Blood glucose levels are managed by insulin. To further evaluate the effect of smoking on glucose metabolism, we quantified serum insulin. The smoking rats exhibited a significantly decreased level of insulin secretion compared with normal control rats (Figure 1C). The administration of melatonin to smoking rats increased insulin secretion and decreased blood glucose. Further, the HbA1c level was also downregulated by melatonin, which demonstrated the therapeutic effect of melatonin on hyperglycemia. Insulin histochemical staining showed that the β cell area was significantly decreased in the islets of smoking rats, whereas the β cell area was increased in the islets of smoking+melatonin rats (Figure 1D,E, *P* < .05). These results confirmed the damaging effects of smoking and protective effects of melatonin on β cells.

### 3.2 Melatonin protects the secretory function and cellular structure of β cells

**Figure 2:**
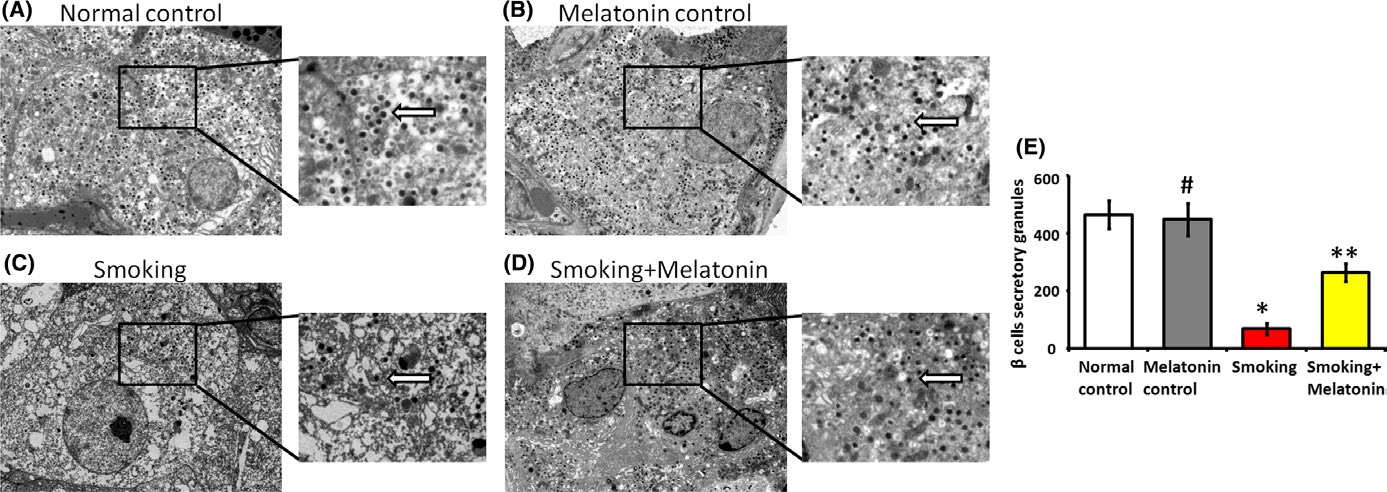
The secretory granules (white arrow) and cytoarchitecture of β cells were examined by electron microscopy. (A) The β cells of normal control rats: abundant secretory granules and a clear cellular structure are evident. (B) The β cells of melatonin control rats: abundant secretory granules and a clear cellular structure are evident. (C) The β cells of smoking rats: the secretory granules are sparse, and the faint cytoarchitecture can be observed. (D) The β cells of smoking rats with intraperitoneally injecting melatonin: compared with the β cells of smoking rats, increased number of secretory granules and distinct cytoarchitecture can be observed. (E) The column diagram shows the number of secretory granules in the different groups. 20 000× magnification. #*P* > .05 melatonin control vs normal control, *P < .05 smoking vs normal control, **P < .05 smoking + melatonin vs smoking

Secretion is the most important function of β cells, and the normal cytoarchitecture of β cells enables this secretory function. The electron microscopy images showed that normal β cells displayed a distinct structure filled with abundant secretory granules (Figure 2A,B). After exposure to smoking, β cells displayed indistinct organelles and sparse secretory granules (Figure 2C), indicative of reduced activity and secretory function. Remarkably, melatonin administration significantly increased the number of secretory granules in β cells, consistent with overall improved organelle appearance (Figure 2D). These data directly demonstrate the protective effects of melatonin on β cells exposed to smoking.

### 3.3 Melatonin decreases the inflammatory reaction and ROS production in the islets of smoking rats

**Figure 3:**
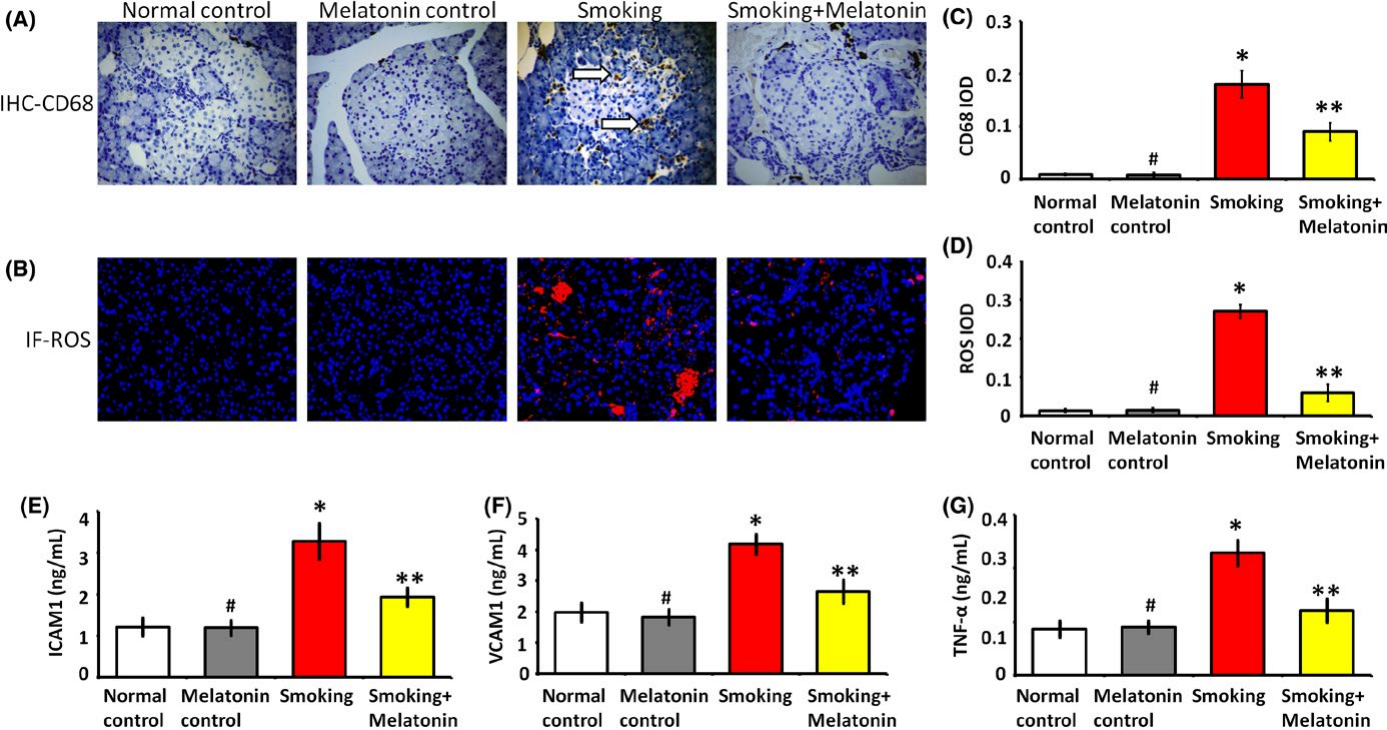
Inflammation and ROS production in islets. (A) In immunohistochemistry (IHC) images, the white arrow shows the infiltration of CD68 inflammatory cells into islet. (B) In immunofluorescence (IF) images, the red fluorescence shows ROS production in islets. (C and D) These histograms show the quantification of integrated optical density (IOD) from CD68 and ROS images, respectively. (E, F and G) The levels of inflammatory factors, intercellular adhesion molecule‐1 (ICAM1), vascular cell adhesion molecule‐1 (VCAM1), and tumor necrosis factor‐α (TNF‐α) in islets suspension, respectively. #*P* > .05 melatonin control vs normal control, *P < .05 smoking vs normal control, **P < .05 smoking + melatonin vs smoking

Tissue dysfunction often results from inflammatory cell infiltration and ROS production. As a marker of monocytes/macrophages, CD68 is expressed in locally infiltrating inflammatory cells. Our results showed that smoking caused the infiltration of CD68‐positive cells (Figure 3A‐C). Furthermore, the infiltrating CD68 inflammatory cells induced ROS production in the islets of smoking rats (Figure 3B‐D). In contrast, melatonin protected the islets from the infiltration of CD68 inflammatory cells and decreased the production of ROS. Besides, the inflammatory factors, intercellular cell adhesion molecule‐1 (ICAM1), vascular cell adhesion molecule 1 (VCAM‐1), and tumor necrosis factor‐α (TNF‐α), that are typically expressed on the activated inflammatory cells, significantly upregulated in the smoking group compared to the normal control group (Figure 3E‐G). Accompanying with the downregulation of CD68 inflammatory cell infiltration, the melatonin administration attenuated the over‐expression of inflammatory factors in smoking rats (Figure 3E‐G). As the inflammatory reaction and ROS production are associated with apoptosis, we further analyze the function of β cells in different groups.

### 3.4 Melatonin attenuates the smoking‐induced β cell apoptosis

**Figure 4:**
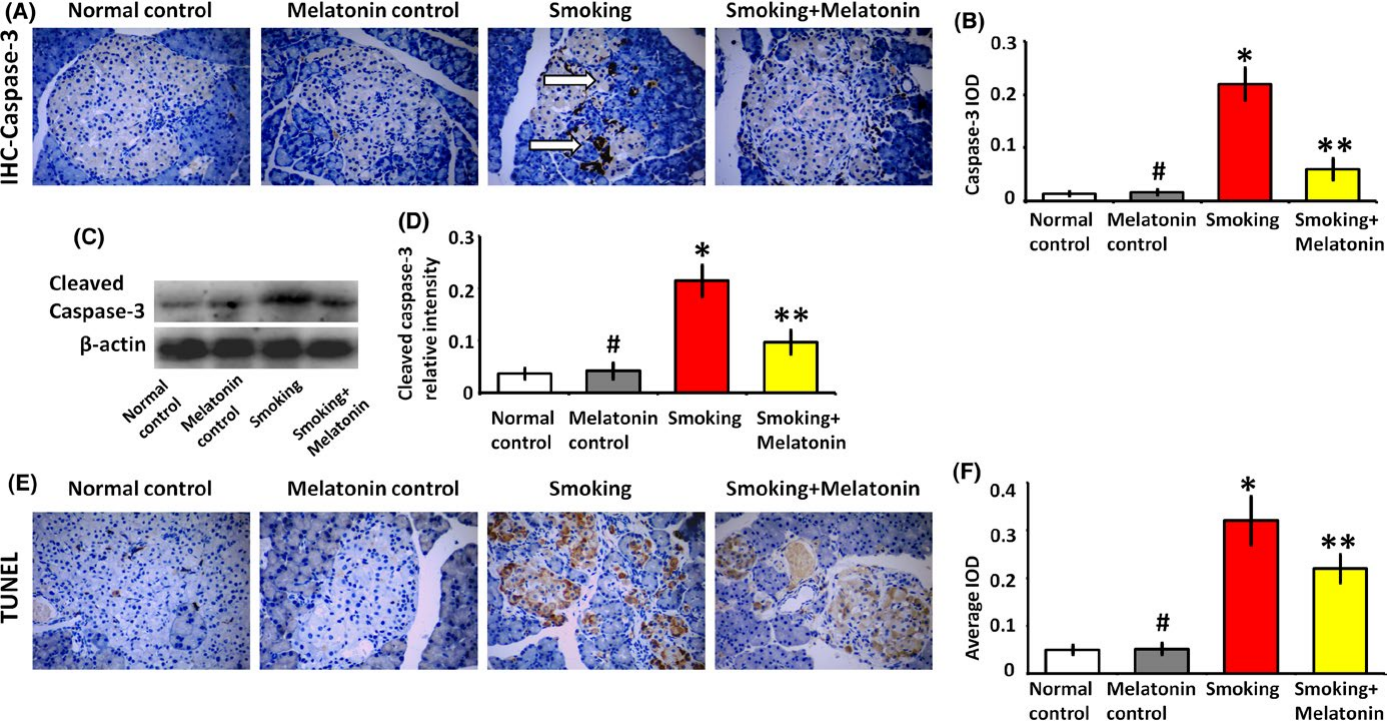
Melatonin attenuates the smoking‐induced apoptosis. (A) In immunohistochemistry (IHC) images, the white arrow shows the expression of the apoptotic caspase‐3 in the islet. (B) The average integrated optical density (IOD) value for caspase‐3 in the IHC image. (C) Western blot analysis of cleaved caspase‐3 expression in β cells, which were cultured in the medium with 5% rat serum of different groups for 24 h. (D) Quantitative analysis of cleaved caspase‐3 expression. (E and F) TUNEL assay to detect apoptotic cells in islets. #*P* > .05 melatonin control vs normal control, *P < .05 smoking vs normal control, **P < .05 smoking+melatonin vs smoking

The hyperglycemic effect of smoking is mainly due to the promotion of apoptosis in the β cells. As a critical executioner of apoptosis, caspase‐3 was significantly expressed in the islets of smoking rats (Figure 4A,B), which demonstrated the execution phase of cell apoptosis. Caspase‐3 activation requires the cleavage of caspase‐3 that triggers the hallmark caspase cascade characteristic of the apoptotic pathway. We further analyzed the apoptotic effect of smoking on β cells in vitro. The expression of cleaved caspase‐3, that is the activated form of caspase‐3, was significantly increased in the β cells of smoking group (Figure 4C,D). In vivo, we also confirmed the smoking‐induced apoptosis of islets in smoking rats, while melatonin attenuated the smoking‐induced β cell apoptosis (Figure 4E,F).

### 3.5 Melatonin preserves hepatic glycogen synthesis

**Figure 5:**
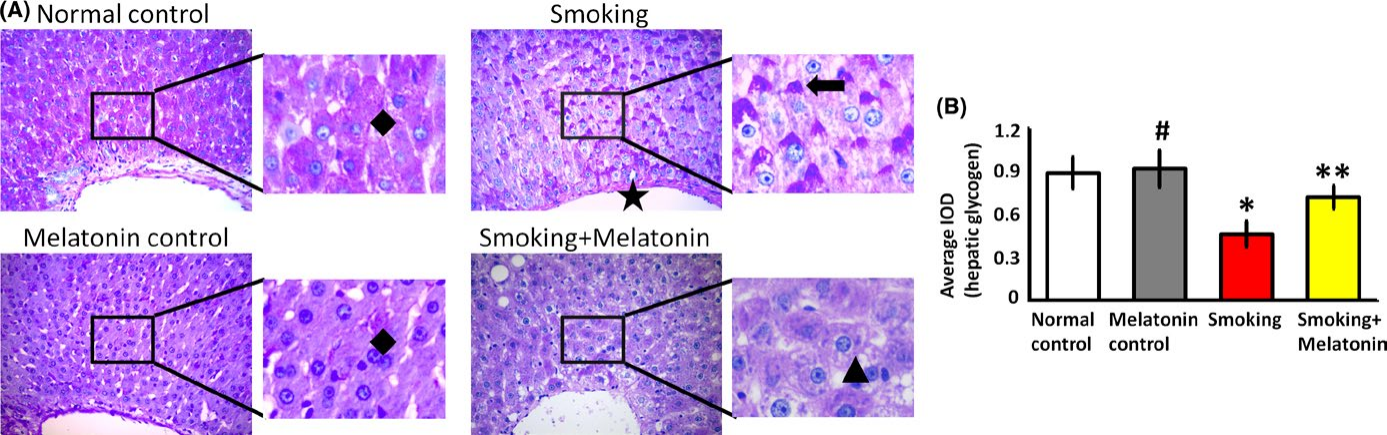
The amount of glycogen in livers. (A) In Periodic acid‐Schiff stain (PAS) images, the staining area shows the glycogen in livers (black rhombuses show that hepatic glycogen homogeneously distributes in hepatocytes; black star shows the central vein; black arrow shows that the amount of hepatic glycogen in hepatocytes of smoking rats decreases and shifts away from the central vein side; black triangle shows that the glycogen deposition is increased by melatonin.). (B) The average integrated optical density (IOD) value for PAS. #*P* > .05 melatonin control vs normal control, *P < .05 smoking vs normal control, **P < .05 smoking+melatonin vs smoking

Hepatic glycogen, which is synthesized by glucose in liver, exerts a hypoglycemic effect during blood glucose metabolism. Further, hepatic glycogen reflects the metabolic balance between glucose and insulin. Accompanied by pancreatic islet dysfunction, smoking disturbed gluconeogenesis in the liver. As shown by glycogen staining (Figure 5), smoking significantly decreased the amount of glycogen in smoking rats, whereas melatonin increased glycogen deposition. As shown in the partial enlarged detail of Figure 5, the amount of hepatic glycogen in hepatocytes of smoking rats decreased and shifted away from the central vein side (black arrow), whereas the glycogen deposition was increased by melatonin and homogeneously distributed in hepatocytes. The restoration of hepatic glycogen ameliorated hyperglycemia.

**Figure 6:**
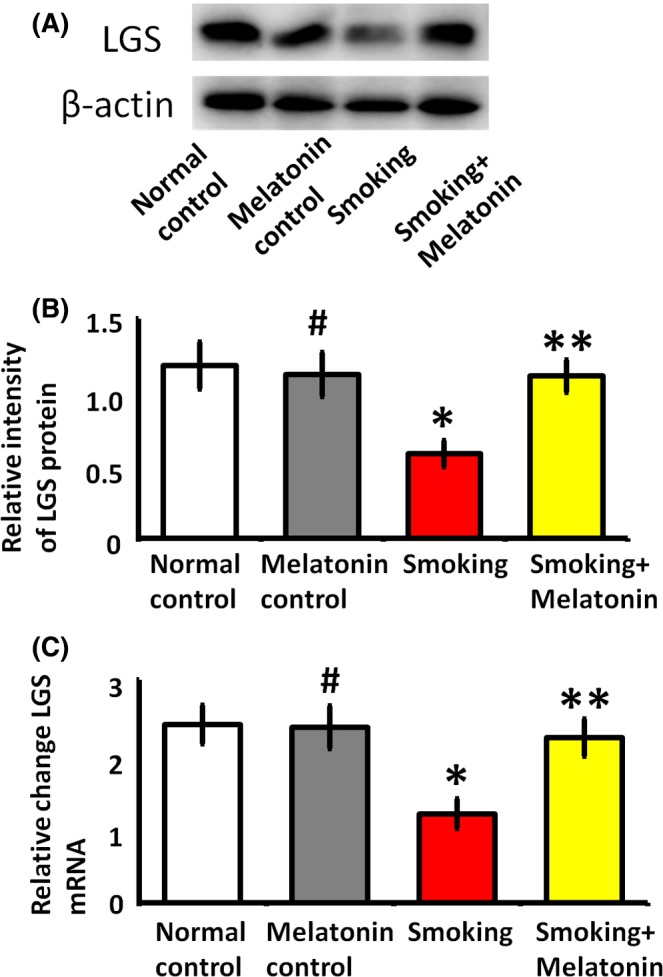
The expression of glycogen synthase (LGS) in hepatocytes. The hepatocytes were cultured in the medium with 5% rats serum of different groups for 24 h. (A) Western blot analysis of LGS expression. (B) Quantitative analysis of LGS expression. (C) Quantitative real‐time PCR analysis of LGS gene transcription. #*P* > .05 melatonin control vs normal control, *P < .05 smoking vs normal control, **P < .05 smoking + melatonin vs smoking

High levels of serum glucose can be cleared from the blood by LGS, which converts glucose into glycogen in the liver. We found that smoking reduced LGS protein expression and mRNA translation (Figure 6). As glycogen synthesis was found to be aberrant in smoking rats, we further investigated whether melatonin preserves the expression of LGS. The results showed that LGS protein expression and mRNA translation were significantly increased by melatonin administration in smoking rats (Figure 6); this effect likely explains the hypoglycemic mechanism of melatonin in the liver.

### 3.6 MT2 is responsible for the effect of melatonin on β cells and hepatocytes

**Figure 7:**
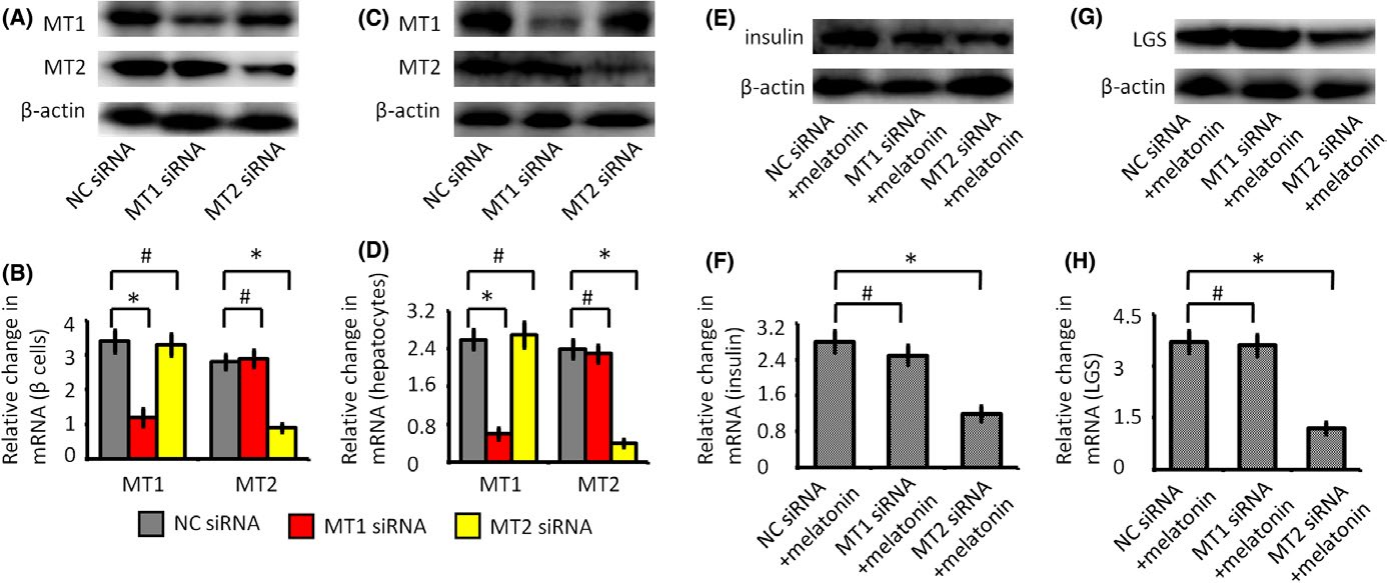
The effects of melatonin receptors on glucose metabolism. (A) Western blot analysis of melatonin receptors (MT1 and MT2) expressions in β cells. (B) Quantitative real‐time PCR analysis of MT1 and MT2 gene transcription in β cells. (C) Western blot analysis of MT1 and MT2 expressions in hepatocytes. (D) Quantitative real‐time PCR analysis of MT1 and MT2 gene transcription in hepatocytes. (E) Western blot analysis of insulin expressions in β cells. (F) Quantitative real‐time PCR analysis of insulin in β cells. (G) Western blot analysis of liver glycogen synthase (LGS) expressions in hepatocytes. (H) Quantitative real‐time PCR analysis of LGS gene transcription in hepatocytes. NC siRNA group, cells were transfected with negative control siRNA; MT1 siRNA group, cells were transfected with MT1 siRNA; MT2 siRNA group, cells were transfected with MT2 siRNA; NC siRNA + melatonin group, cells were transfected with negative control siRNA, and then cultured with melatonin for 24 h; MT1 siRNA + melatonin group, cells were transfected with MT1 siRNA and then cultured with melatonin for 24 h; MT2 siRNA+melatonin group, cells were transfected with MT2 siRNA and then cultured with melatonin for 24 h. #*P* > .05, *P < .05

As melatonin has a positive effect on glucose metabolism, the melatonin receptors, MT1 and MT2, may mediate the insulin secretion in β cells and LGS expression in hepatocytes. To further examine the precise roles of MT1 and MT2, siRNA‐mediated knockdown was used to abrogate the expression of MT1 and MT2, respectively (Figure 7). After inhibiting the expression of MT2, the effect of melatonin on insulin and LGS expression were significantly inhibited. In contrast, the decrease in MT1 did not affect the effect of melatonin on β cells and hepatocytes. Functional analysis showed that the MT2 receptor was more important to glucose metabolism.

## 4 DISCUSSION

### 4.1 Smoking disturbs glucose metabolism and induces hyperglycemia

**Figure 8:**
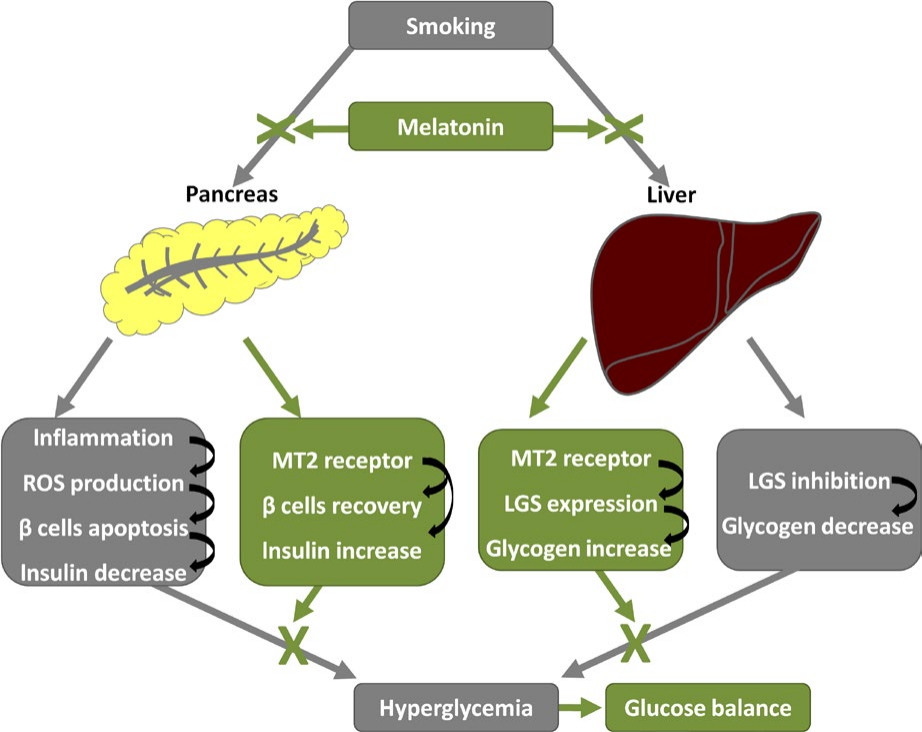
Diagram represents the protective function of melatonin against smoking‐induced hyperglycemia. The gray blocks and lines represent the effects of smoking. The green blocks and lines represent the effects of melatonin

Smoking has detrimental effects on health. Our study demonstrated that smoking disturbed glucose metabolism and induced hyperglycemia in rats (Figure 8). Further, the elevation of hemoglobin A1c reflected the progression of hyperglycemia in smoking rats. The disturbance of glucose metabolism raised the question of whether smoking affects insulin secretion. To address this issue, we analyzed the effect of smoking on β cell function. The results showed that smoking significantly decreased serum insulin and islet mass in rats. Moreover, smoking significantly induced the expression of apoptotic caspase‐3 and decreased the number of secretory granules in β cells. As an important factor in glucose homeostasis, the expression of LGS was also impaired by smoking, which decreased hepatic glycogen deposition and contributed to the development of hyperglycemia. Given that smoking affects multiple aspects of glucose metabolism and causes hyperglycemia, it is important to develop therapeutic approaches and investigate the underlying mechanisms.

Smoking is primarily responsible for an increase in inflammatory factors, leading through this mechanism to β cell dysfunction and increasing diabetes risk. In the process of smoking‐induced islets injury (Figure 3), there was obvious CD68 inflammatory cell infiltration, which caused β cell dysfunction. Our previous study also demonstrated that CD68 inflammatory cells attached to endothelium in diabetes and balloon‐injury carotid artery model, and then induced the process of atherosclerosis.^24^, ^25^ In smoking, we proved that the infiltration of CD68‐positive cells is the trigger mechanism related to hyperglycemia. The infiltration CD68 cells are mainly monocytes/macrophages in inflammatory tissues and can express the excessive inflammatory cytokines.^26^, ^27^ Therefore, led by the inflammatory infiltration of CD68‐cells, smoking caused the hyperglycemia in rats. Our randomized controlled trial and experimental animal study also demonstrated that smoking can induce the elevation of inflammatory factors.^9^ In smoking, the composite effects of inflammatory cytokines have been found to predict diabetes risk and associate with impaired fasting glucose.^28^

Epidemiologic studies reveal that levels of inflammatory cytokines increase in subjects with diabetes and associate with insulin resistance.^29^ TNF‐α is mainly produced by monocytes/macrophages and is involved in insulin resistance by decreasing the expression of insulin, increasing free fatty acids, and downregulating peroxisome proliferator‐activated receptor γ, an insulin‐sensitizing nuclear receptor.^30^, ^31^ The inflammatory factors, ICAM1 and VCAM1, are over‐expressed in inflammatory cells by smoking, which typically mediate the transmigration of CD68 inflammatory cells into islet and the dysfunction of β cells in positive feedback. They also play the role in the development of microcirculation disorder that can decrease the blood flow of islet and deteriorate the dysfunction of β cells.^25^ Upon smoking stimulation, these inflammatory responses also can be aggravated by TNF‐α. In our study, the CD68 inflammatory cells infiltration indicated that smoking might contribute to insulin resistance and hyperglycemia through increasing inflammatory cytokines levels. Additionally, the oxidative stress injury also occurs in the smoking‐induced hyperglycemia. CD68 cell infiltration is significantly associated with the ROS production (Figure 3), which can cause oxidative damage and eventually lead to β cell apoptosis (Figure 4). Finally, the inflammation and oxidative stress contribute to the pathogenesis of diabetes. It is a key point to attenuate inflammation in therapeutic regimen. Although melatonin is reported to decrease the over‐expression of inflammatory cytokines,^9^ little information is available regarding whether melatonin plays a protective role in decreasing inflammation and reducing oxidative stress in relation to smoking‐induced hyperglycemia.

### 4.2 Melatonin regulates glucose metabolism and ameliorates hyperglycemia

Melatonin has received attention as a potential therapeutic agent maintaining blood glucose homeostasis,^3^, ^4^ and the influence of melatonin on smoking‐induced hyperglycemia is less well documented. Our study demonstrated that melatonin could normalize insulin secretion and blood glucose in smoking rats (Figures 1 and 2). The recovery of β cell area in insulin IHC images also indicated that melatonin maintains glucose homeostasis and decreases the risk of hyperglycemia in smoking rats (Figure 1). Previous studies also suggested that reduced melatonin level or mutations of the melatonin receptors were associated with increased risk of developing diabetes.^32^, ^33^, ^34^ Further, melatonin administration reduced HbA1c levels and improved glucose tolerance (Figure 1). Another study reported a significant improvement of glucose tolerance in obese mice treated with melatonin.^35^ Altogether, melatonin exerts a positive influence on glucose homeostasis and insulin secretion. We further analyzed the mechanisms by which melatonin improves glycemic control.

### 4.3 Melatonin protects β cells functions

Our results demonstrated that melatonin protects β cell function and insulin secretion, which are vulnerable to smoking‐related inflammation and oxidative stress. As shown in IHC images (Figures 1 and 4), melatonin restored β cell area and inhibited β cell apoptosis in the smoking rats. These data support the notion that melatonin attenuates β cell damage and improves glucose homeostasis. In tissues, inflammatory infiltration often indicates dysfunction and apoptosis. As a marker of inflammatory cells, CD68 is useful in assessing inflammatory infiltration.^36^ In the islets of smoking rats, there was significant infiltration of CD68‐positive cells (Figure 3). Inflammatory cell infiltration causes the dysfunction of β cells in smoking rats. Furthermore, CD68 infiltrating cells trigger ROS production in the mitochondrial electron chain transport,^37^ which subsequently induces oxidative stress injury and eventually causes dysfunction and apoptosis of β cells. As activated in β cells exposed to smoking, caspase‐3 eventually executes apoptosis either by extrinsic or intrinsic pathway and plays a central role in the execution phase of β cell apoptosis. In addition to its chronobiotic activity, melatonin has anti‐inflammatory and antioxidative effects,^38^, ^39^, ^40^ ranging from the direct scavenging of ROS to the regulation of antioxidant enzymes, such as superoxide dismutase, catalase, and glutathione peroxidase.^41^ Also, melatonin can promote the expression of glucose transporter, glucose transporter type‐4, which can be inhibited by oxidative stress.^42^ Our previous study has shown that melatonin could attenuate inflammatory and oxidative injury caused by cigarette smoking and preserved vascular function.^9^ In the study, we confirmed that melatonin protects β cell functions from CD68 cell‐related ROS injury.

### 4.4 Melatonin preserves the glycogenesis of liver

The liver plays an important role in normalizing blood glucose by converting glucose into hepatic glycogen.^43^, ^44^ In contrast to insulin, the action of hepatic glucagon is rapid and short‐lived. Apart from decreasing insulin secretion, smoking impaired the accumulation of hepatic glycogen (Figure 5), thereby contributing to the development of hyperglycemia. Additionally, the smoking‐mediated decrease in insulin secretion weakened the capacity for glucose utilization in the liver. However, melatonin increased glycogen deposition, which was beneficial for the glycemic control. Therefore, these results indicate that the ability to synthesize hepatic glycogen synthesis may be involved with glucose metabolism in smoking rats.

As the key enzyme in hepatic glycogen synthesis, LGS participates in the regulation of blood glucose. In our study, melatonin administration recovered the expression of LGS in smoking rats (Figure 6). Previous studies also showed that the overproduction of LGS in rats was an effective method for improving glucose tolerance, owing to its capacity to enhance glucose storage.^14^, ^45^ Furthermore, the increased activity of LGS potentially enhances insulin sensitivity in the liver,^46^ which may explain the insulin‐sensitizing effect of melatonin in smoking rats. Additionally, in our previous study, transcription factor nuclear erythroid 2‐related factor 2 (Nrf2) is the key factor in protective properties of melatonin.^10^ Through Nrf2 signaling pathway, melatonin is important for the coordinated upregulation of genes in response to oxidative stress. As melatonin is a cytoprotector in response to smoking, Nrf2 may be involved in the transcriptional activation of LGS. Together, our results demonstrate that LGS is involved in mediating the protective effects of melatonin, which improves glucose metabolism in smoking rats. Therefore, this study shows that melatonin protects against smoking‐induced hyperglycemia and may provide a novel therapeutic strategy for treating hyperglycemia.

### 4.5 Melatonin regulates glucose metabolism through the MT2 receptor

Many of melatonin's biological effects are mediated by the activation of melatonin receptors.^4^, ^18^, ^19^ The effects of melatonin are mediated by two receptors, which are expressed in a function‐specific fashion.^47^, ^48^ The melatonin receptors of β cells positively regulate cellular functions and affect insulin secretion in the subtype‐dependent mode.^17^, ^49^ Our study demonstrated that MT2 is responsible for the effect of melatonin on insulin secretion (Figure 7). Additionally, it is shown that melatonin promotes LGS expression in liver. Similarly, MT2 was highly expressed on hepatocytes and was responsible for the expression of LGS (Figure 7). Therefore, melatonin interacts with MT2 receptors, functionally ameliorating the smoking‐induced disturbance of glucose metabolism. Previously, melatonin was shown to act through G‐coupled MT2 receptors to stimulate phospholipase C and inositol triphosphate activity and thus regulate glucose metabolism.^20^, ^50^ As both melatonin receptors are expressed in islet and liver, the precise roles of MT1 and MT2 remain to be elucidated. Additionally, the mechanisms of the melatonin effects can be roughly distinguished in receptor‐dependent and receptor‐independent. The properties of decreasing CD68 inflammatory infiltration and scavenging ROS, that is the melatonin's anti‐inflammation and antioxidant activities, should be attributed to receptor‐independent. However, MT2 receptor was also reported to reduce leukocytes rolling and adhesion and might also explain the anti‐inflammatory effect of melatonin.^51^ Taken together, these results support the hypothesis that protective effects of melatonin on glucose balance of smoking rats are at least partly mediated by MT2 receptor.

Apart from the high‐affinity receptors MT1 and MT2, it is reported that the protective properties of melatonin may have a significant effect on the potential role of the retinoic acid receptor‐related orphan receptor α (RORα), which participates in the gene transcription and plays a critical role in the regulation of many physiological processes, including cell differentiation, metabolic and circadian activities.^52^, ^53^ RORα transcriptional activity can be modulated by melatonin.^54^, ^55^ It is reported that melatonin could affect DNA‐binding activity, expression of RORα‐regulated genes via modulation of the Ca^2+^/Calmodulin kinases signaling pathway.^56^ The functional link between melatonin protective properties and RORα signaling pathway appears to be justified by the observed pleiotropic phenotypic effects of LGS expression and insulin excretion.

### 4.6 The clinical implications

As the research proved, there are close relationships between smoking and diabetes. We presented the mechanism explanation of clinical phenomenon, which provide strong evidence for quitting smoking among people suffering from diabetes. In the smoking‐induced β cell dysfunction, insulin resistance, and hepatic glycogen synthesis disorder may be the target of treatment. And then, the study displayed the protective mechanism and therapeutic potential of melatonin in smoking‐induced hyperglycemia. In smoking, melatonin effectively can prevent inflammation and oxidative damage.^57^, ^58^ As oxidative stress plays an important role in smoking‐induced injury, melatonin supplementation reduces hyperglycemia and improves the antioxidant status.^57^ Melatonin effectively can protect cells from mitochondrial ROS, and the role of melatonin in preventing mitochondrial dysfunction might be important.^58^ Knowing that optimal glycemic control is usually an issue for diabetic patients with smoking,^59^ melatonin administration may be considered as an effective regimen to the current treatment.

There is no drug therapy for preventing glucose metabolism disturbance from smoking. Therefore, melatonin should be a potential therapeutic regimen, which has a high safety profile and differs from other pharmaceutical agents that inhibit glucose intake. In particular, melatonin can improve insulin sensitivity through promoting mitochondrial functions of subcutaneous fat and skeletal muscle 46, 60, 61 and ameliorate hepatic steatosis via increasing hepatic glycogen content in the liver,^62^ which may benefit the obesity‐combined smokers. What's more, reduced levels of melatonin were described in diabetes.^63^, ^64^ Therefore, melatonin administration can improve the current treatment and help to overcome the hyperglycemia effects that are not always alleviated by the traditional therapy. Notably, more studies are needed to identify the appropriate duration of treatment and dose of administration. The results of this study encourage a prospective clinical trial to evaluate the therapeutic potential of melatonin in the smoking‐induced hyperglycemia.

### 4.7 Research prospects

There are also some limitations to our study. In islets, CD68 cell infiltration and ROS production damage β cells and decrease insulin secretion. However, abundant circulating hemopoietic cells are present in hepatic sinusoids,^65^ which make it impossible to detect CD68 cell infiltration and ROS production. Further studies of the effect of melatonin on the ROS‐related mitochondrial electron chain transport are needed. Moreover, the present in vitro results demonstrate that melatonin acts through the MT2 receptor to regulate the blood glucose metabolism. As MT2 interacting with MT1 in vivo,^66^, ^67^, ^68^ additional study is required to delineate the precise function of MT2 using gene knockout rats. To homogenize the amount of smoking, we carried out the present study in rats. While this study lacks epidemiologic data, our previous double‐blind randomized controlled trial involving 63 participants (approved by the Ethical Committee of Peking Union Medical College Hospital, No.JS‐863, and registered in Clinical Trials.gov, “Melatonin in smoke induced vascular injury,” No. NCT02591238) demonstrated that administering melatonin to smokers lowered their fasting blood glucose levels after 2 weeks of treatment (4.79 ± 1.05 mmol/L vs 5.45 ± 1.69 mmol/L, *P* = .09).^10^ The effects of smoking on glucose metabolism may include both insulin secretion and resistance, and assessment using a homeostasis model should be performed in the smoking population. An additional large‐scale epidemiological study is warranted to explore the clinical impact of these findings and investigate the effects of melatonin on glucose metabolism in smokers.

## 5 CONCLUSIONS

In conclusion, smoking is thought to affect glucose metabolism by impairing β cell function in the pancreas and inhibiting LGS expression in the liver. Melatonin preserves insulin secretion and liver glycogen synthesis in smoking rats. Notably, MT2 may be responsible for the effects of melatonin on the regulation of glucose metabolism. These data provide strong evidence for quitting smoking among people suffering from diabetes. And, the study provides a mechanistic basis for therapeutic regimens targeting abnormal glucose metabolism in smokers. Melatonin may be a plausible candidate drug for treating smoking‐induced hyperglycemia.
